# Intellectual Sports

**Published:** 1884-05

**Authors:** 


### INTELLECTUAL KINDS OF SPORT.

A WELL-KNOWN divine whose long and wide connection with the youth of his country, makes his opinion interesting, and who is himself no fisherman, is fond of affirming that no one who is addicted to fishing can be intellectually or morally hopeless, as may be the case with the most consistent devotees of the more gregarious and showy sports; that the mere fact of his being an angler is evidence in his favor; and that no irretrievably dull soul, no one without some mental stamina or food for reflection, could stand his own company, through the long solitary days by lake or river, that the angler's life entails.

